# The Thermal and Mechanical Behaviour of Wood-PLA Composites Processed by Additive Manufacturing for Building Insulation

**DOI:** 10.3390/polym15143056

**Published:** 2023-07-16

**Authors:** Anis Bahar, Ameur El Amine Hamami, Ferhat Benmahiddine, Sofiane Belhabib, Rafik Belarbi, Sofiane Guessasma

**Affiliations:** 1 Department of Mechanical Engineering, IUT Nantes, Nantes Université, Oniris, CNRS, GEPEA, UMR 6144, F-44000 Nantes, France; anis.bahar@univ-lr.fr (A.B.); sofiane.belhabib@univ-nantes.fr (S.B.); 2 INRAE, UR1268 Biopolymères Interactions Assemblages, F-44300 Nantes, France; 3 LaSIE, UMR 7356 CNRS-La Rochelle Université, Avenue Michel Crépeau, CEDEX 01, F-17042 La Rochelle, France; ahamami@univ-lr.fr (A.E.A.H.); ferhat.benmahiddine@builders-ingenieurs.fr (F.B.); rbelarbi@univ-lr.fr (R.B.); 4 Builders Lab, Builders Ecole d’Ingénieurs, ComUE NU, 1 Rue Pierre et Marie Curie, F-14610 Epron, France; 5 Department of Architecture, Canadian University Dubai, City Walk, Dubai P.O. Box 415053, United Arab Emirates

**Keywords:** wood-PLA filament, fused filament fabrication, thermal properties, tensile performance, microstructure

## Abstract

This study was aimed at considering the potential of wood-based composites processed using additive manufacturing as insulators in the building sector. A polylactic acid blend with 30% wood particles was used as a feedstock material in fused filament technology. Its thermal and mechanical properties were determined for various processing conditions, including printing temperature and infill rate. The results showed a minor contraction in its tensile performance as a result of the printing process. The printing temperature had a negligible effect on its stiffness and a limited influence on the other engineering constants, such as the tensile strength and ultimate stress. The thermal properties of printed structures have been found to significantly depend on the infill rate. Although the tested 3D printed wood-PLA material exhibited good thermal properties, which were tuneable using the printing conditions, its performance was still 38% to 57% lower compared to insulators such as the glass wool of the synthetic foams used in the building sector.

## 1. Introduction

Providing up to 40% of the global energy consumption and 30% of greenhouse gas emissions, the building sector is considered to be one of the most energy-consuming sectors. Furthermore, the report presented by the Intergovernmental Panel on Climate Change (IPCC) presented alarming results on current and future climate change [1]. Within this context, the new French RE2020 regulation [2] introduced new requirements aimed at limiting the primary energy consumption and reducing the environmental impact of building components. One of the ways to respond to this alarming observation is to use construction materials with a low environmental footprint, such as bio-based and/or geo-based materials [3,4,5].

The study conducted by P. Ricciardi et al. [6] on the thermal and environmental behaviour of several materials developed with a polyurethane adhesive and industrial and agricultural waste showed that samples composed of cork, coffee flakes, and rice husks presented good thermal performances and a reduced environmental footprint. Although the incorporation of biobased materials adds more complexity to the interpretation of mass and transfer properties, promising insulating properties can be achieved if a precise control of the microstructure is achieved. This local control can be driven by new material-processing technologies, such as the additive manufacturing route [7,8], where the sourcing of the material combined with an appropriate material architecture can lead to a substantial reduction in the energy consumption in the building sector, through more innovative material designs [9]. Fused filament fabrication (FFF) is one of the additive manufacturing processes that is extensively used to produce polymeric parts [10]. In fused filament technology, the mastering of the rheological behaviour of the feedstock material is the key to achieving rapid rigidification after the cooling down of a filament heated above its glass transition temperature [11]. Filament solutions based on biosourced material have been considered in fused filament technology [12]. The most common examples that can be found in the literature refer to polylactic (PLA) filaments. The environmental impact of PLA is positive when considering the sourcing of the material without transport costs. However, the LCA (Life Cycle Analysis) of PLA can be subject to debate depending the availability of a short supply chain. Thanks to governmental initiatives and new regulations, this supply chain issue is being solved, promoting large expectations about the environmental footprint of PLA or PLA-based composites compared to synthetic insulator materials such as glass wool, polystyrene, or polyurethane foams. In addition, a recent study conducted by Ulkir [13] showed that the energy consumption of PLA is the lowest among the synthetic materials used as a feedstock in fused filament technology, such as ABS and PETG.

A new trend in filament development has been witnessed, which is related to the use of biobased composites as feedstock materials [14]. The idea behind this is that PLA’s production and environmental cost can be lowered using fused filament fabrication, when PLA is blended with a second phase such as wood particles, as this type of filler is cheaper, environmentally friendly, and exhibits a lower thermal conductivity, up to two times that of PLA. Thus, most research has focused on the use of a PLA matrix reinforced by biobased fillers such as wood, flax, or hemp fibres [15,16,17]. For instance, Liu et al. [18] compared the performance of wood with varieties of feedstock materials such as ceramic, metal, and carbon blended with PLA. The authors concluded negative roles of wood and chopped carbon fibres compared to ceramic, copper, and aluminium counterparts in terms of their reinforcing effect in a PLA matrix. They also attributed the lower performance of the wood-PLA filament to the defects such as a high porosity and lack of compactness. Kananathan et al. [19] conducted a more focused study on wood-PLA composites by considering the role of the printing parameters. The authors showed that the infill rate and pattern significantly affected the mechanical properties under tensile, flexural, and compression testing conditions. Tao et al. [20] formulated composite filaments based on a PLA matrix and wood flour. The authors showed that the overall ranking of the printed samples from the woody material was lower compared to the ones printed only by the PLA. Yang et al. [21] reported results on the effect of the printing speed on the morphology and mechanical performance of PLA-wood printed composites. The authors showed that the density of the printed PLA-wood composite decreased with an increase in the printing speed, but surprisingly, this had no effect on the tensile and flexural properties.

An analysis of the literature shows that the research work on PLA-wood composites is rather new and only focuses on their mechanical properties. This literature also shows that the mechanical performance of PLA-wood filaments is not large enough to consider this material for structural applications. However, in building insulation, the requirement for mechanical support is not as large as that for structural materials. A typical example of this is glass wool, which is widely used as a building insulator. This opens up a route for the potential use of wood-PLA composites as rigid insulators if their thermal insulation properties reach the standards required in the building sector. In addition, the use of wood filler in a PLA matrix also makes sense, because wood is known as an excellent insulator in the building sector. However, the thermal properties of wood-PLA have not received much attention. One of the aspects that this study focuses on is the consideration of the additive manufacturing route as a way to reveal the potential of wood-PLA composite for thermal insulation, thanks to the possible control of the airiness of this material.

In this study, the objective was to reveal the potential of using wood-PLA as a rigid insulator in the building sector, for which the local control of the thermal properties is ensured by AM.

In the present study, the analysis of both the mechanical and thermal properties of PLA-wood composites is conducted with the aim of using this material as an insulator in the building sector. The main highlight of this study is that the low mechanical performance of PLA-wood composites does not necessarily oppose their good insulation properties, thanks to two main arguments: insulators do not require a large mechanical performance, and the thermal properties can be adjusted by varying the airiness through the additive manufacturing route. In a typical printed part, the air has the lowest thermal conductivity, of the order of 24 mW/mK. When the density of the material is decreased, for instance through the infill rate, the thermal conductivity is affected. In a typical wood-PLA filament, PLA has a thermal conductivity of 183 mW/mK, while that of wood is in the range of 100–200 mW/mK. Creating a porous structure using AM is a way of decreasing the thermal conductivity of the printed part. The process-induced porosity also contributes to these thermal properties. This develops as a result of the discontinuity of the material during the building process. In fused filament technology, material continuity is only ensured in one direction [22]. This leaves two main directions in which a small amount of porosity takes place. However, this porosity is not significant enough to tune the thermal conductivity of a printed part. This is why finding suitable parameters, such as the infill rate, to achieve a leverage on the thermal properties of printed structures is crucial.

## 2. Experimental Layout

**Figure 1:**
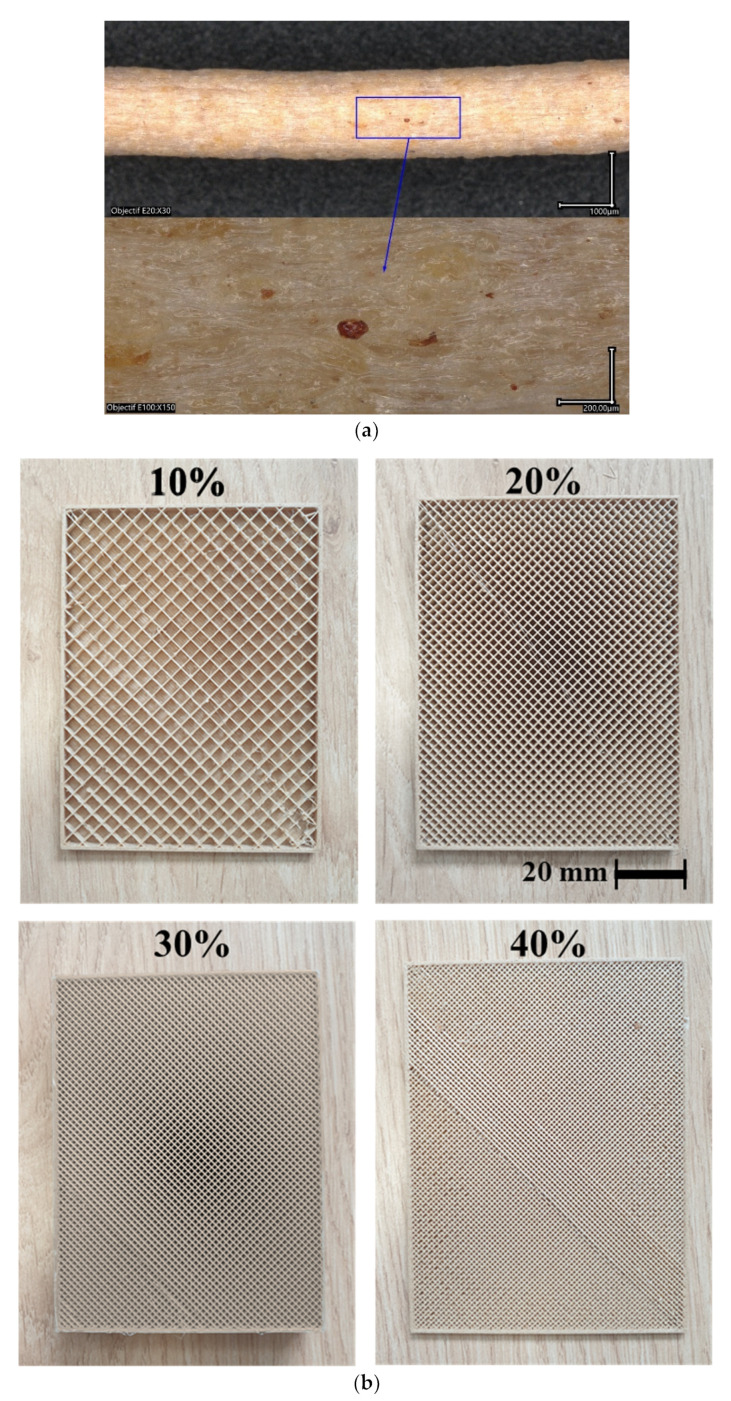
(**a**) Morphology of as-received PHW filament, and (**b**) cross-section views showing 3D printed samples with four levels of infill from 10% to 40% using wood-PLA filament.

The studied composite material (PHW) is a blend composed of PLA-PHA with 30% recycled pinewood particles purchased from ColorFabb (Figure 1a). This amount of 30% corresponds roughly to the percolation limit, above which, issues of flowability start to appear, causing nozzle-clogging problems, especially for nozzles as small as 0.4 mm. The physical properties and recommended printing conditions are given in Table 1.

**Figure 2:**
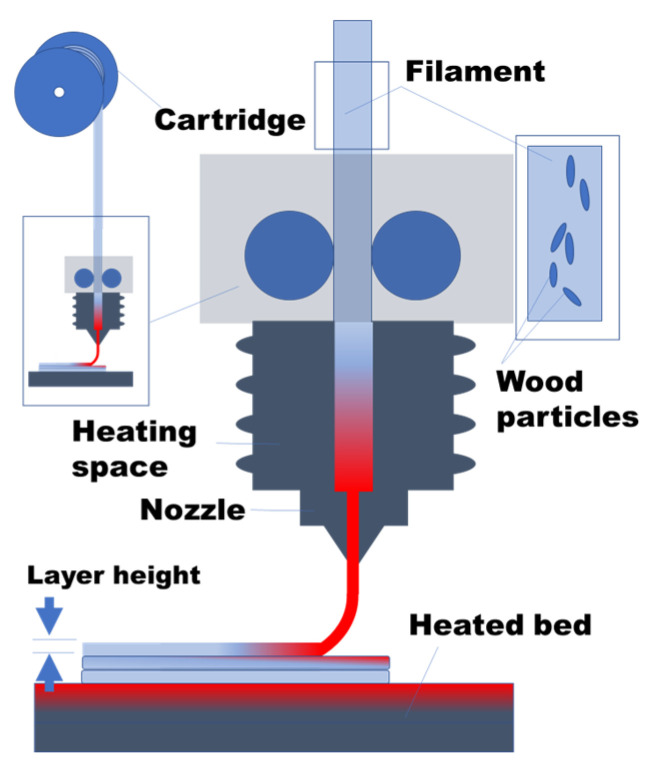
Schematics showing the printing process to achieve 3D printed PHW composites.

The FFF technique is selected for the printing process using a Creality CR10V3 commercial printer (Shenzhen Creality 3D Technology Co., Shenzhen, China), allowing for a printer volume of 300 mm × 300 mm × 400 mm, a printing temperature of up to 260 °C, and a building platform with heating up to 110 °C. Figure 2 illustrates the printing process to achieve the PHW composite material. The experimental protocol adopted in this study is aimed at achieving the regulation of the thermal and mechanical properties of the studied material. The regulation of the mechanical performance is, however, not a hard prerequisite for insulation. According to previous studies by the authors, the printing temperature was found to be a key parameter that influences the mechanical performance, through inter-filament cohesion and porosity reduction [27]. The infill rate is also an influential parameter of thermal conductivity. The larger the amount of air in the structure, the better the thermal insulation properties are. Both the printing temperature and infill rate are considered in the regulation of the thermal and mechanical properties of wood-PLA material.

The geometry of the printed samples is selected depending on the testing protocol constrains. For tensile testing, dogbones of 80 × 20 × 4 mm^3^ are printed, where the sample height is aligned with the building direction. For the thermal analysis, larger samples of 100 × 75 × 20 mm^3^ are printed, where the thickness of the sample is also aligned with the building direction. In order to compare the mechanical performance of the printed samples with the filament’s intrinsic properties, the tensile testing of the PHW wires is performed on samples 40 mm in length and 1.75 mm in diameter. The fixed printing conditions are detailed in Table 2.

In addition to the fixed printing parameters, the printing temperature (T_P_) is adjusted to several levels, namely 200 °C, 210 °C, and 220 °C, while the bed temperature (T_B_) is fixed to one of the two levels, 50 °C and 60 °C. For the thermal analysis, four levels of infill rates are selected (10%, 20%, 30%, and 40%), for which the zigzag pattern is selected (Figure 1b), according to previous work by the research group [27]. For the mechanical testing, an infill rate of 100% is considered and the geometry of the tensile specimens is adjusted according to the norm ISO 527-1/-2 [28].

**Figure 3:**
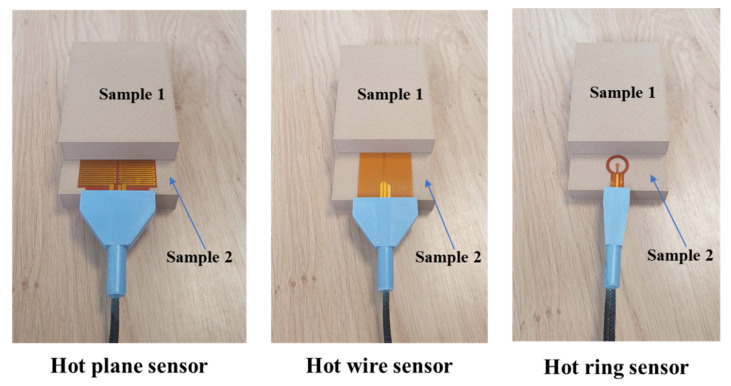
Experimental setup used to measure the thermal properties of 3D printed PHW.

The morphology of the as-received PHW filament is assessed using a numerical optical microscope (Keyence, Osaka, Japon) using different magnification levels, with a pixel size varying from 0.7 µm to 5.1 µm. The thermal properties of printed PHW, namely its thermal conductivity, diffusivity, and effusivity, are determined using a transient hot probe (FP2C, Neotim company, Albi, France) (Figure 3). The thermal conductivity is determined using a hot wire probe measurement consisting of a resistive wire and a thermocouple in an insulating kapton support. The thermal effusivity is assessed using a hot plane probe experiment. Finally, the thermal diffusivity is measured using a hot ring probe experiment. In order to compare the thermal properties of the studied filament with respect to commercially available insulators, the thermal properties are also evaluated for two insulators, including glass wool and synthetic foam. The testing conditions for each material are shown in Table 3. For each measured property, two samples are required to run the experiment. Mechanical testing is conducted on both the wire and printed samples using a universal testing machine (Zwick Roell Group, Ulm, Germany). The tensile conditions are applied using a load cell of 10 kN, a crosshead speed of 5 mm/min, and a large displacement threshold allowing for sample failure. From the force–cross-head displacement signal, the engineering values of Young’s modulus E_Y_, yield stress σ_Y_, tensile strength σ_S_, ultimate stress σ_R_, and elongation at break ε_R_ are extracted and related to the printing conditions. The testing is monitored using a high-speed camera (Phantom V7.3, Photonline, Marly Le Roi, Yvelines, France). The experiments are conducted under low and high image speed recording for the purpose of capturing both the entire tensile events and the rupture sequence at the break point. The frame rate is fixed to 100 fps (frames per second) for a full frame area of 800 × 600 pixels, for which the pixel size is 120 µm.

The fracture patterns are analysed using Scanning Electron Microscopy (SEM) to derive qualitative information about the deformation mechanisms. JEOL JSM-5800 equipment (IMN, Nantes, PDL, France) is used with an operating voltage of 10 kV. An Everhart–Thornley secondary electrons detector is used. Prior to observation, the samples are coated with a 50 nm carbon layer using a Balzers CED 30 evaporator. The magnifications are varied from 50× to 2000× under a typical pixel size varying from 55 nm to 2 µm.

In order to study the thermal transitions in both the filaments and printed structures, a Differential Scanning Calorimetry (DSC)/Thermogravimetric Analysis (TGA) is performed. The experiments are undertaken using DSC3+ equipment (Mettler-Toledo, Zürich, Switzerland) to derive the crystallization, melting, and thermal degradation. DSC is also exploited to obtain the amount of wood filler in the filament. The testing protocol considers the following temperature profile: isotherm stage at 25 °C, maintained for 5 min, followed by a heating stage from 25 °C up to 450 °C, with a heating rate of 5 °C/min. For the extruded filaments, a DSC analysis is performed for all printing temperatures from 200 °C to 220 °C to assess the structural changes affected by the printing process.

## 3. Results and Discussion

### 3.1. Morphology of PHW Filament

**Figure 4:**
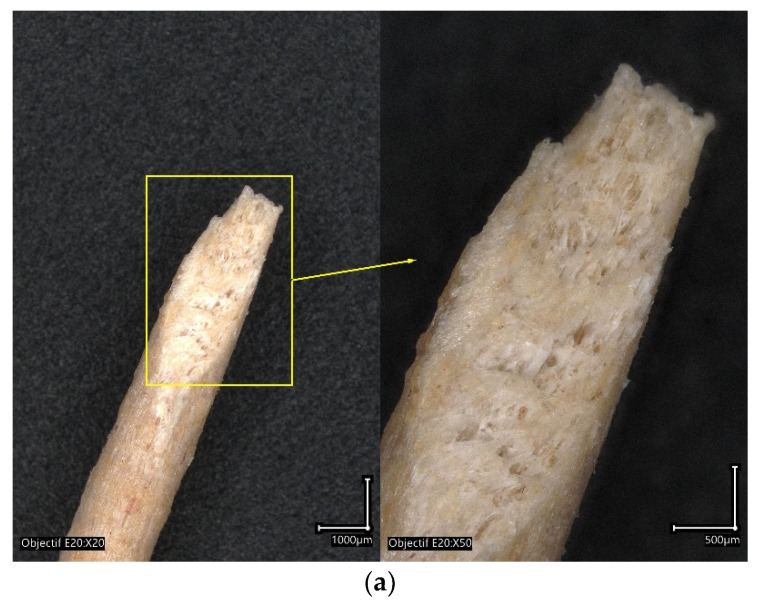

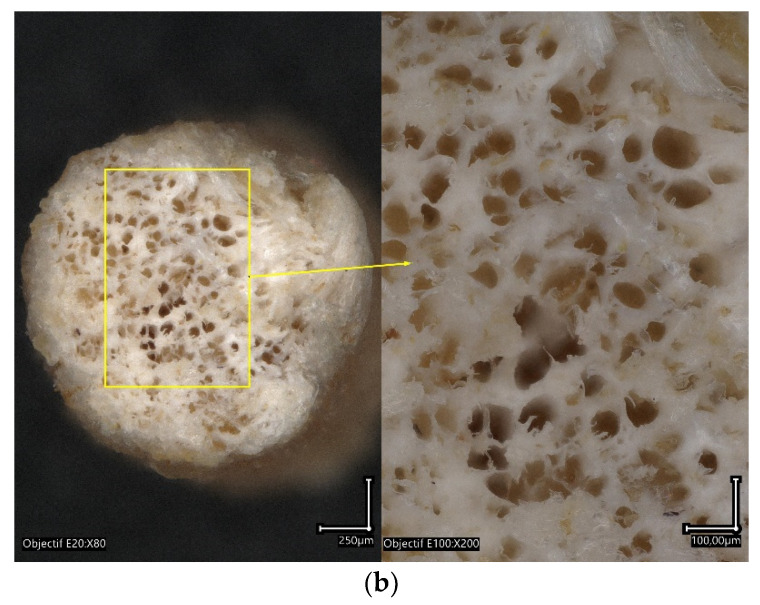
Morphology of as-received PHW filament analysed using optical microscopy, (**a**) oblique cut, and (**b**) magnified view of the filament cross-section.

Optical micrographs show the main characteristics of the as-received PHW. A rough surface finish is the main feature observable at a low magnification (Figure 4a). The colour contrast highlights the presence of wood particles on the surface of the filament, but their surface proportion seems to be lower compared to the supplier reported data of 30%. A cross-section view shows the presence of inner porosity (Figure 4b), which can be regarded as globular and homogeneous, distributed across the section. The observation of a fracture pattern after filament pull-out shows the voids left by the wood particle extraction combined with the intrinsic porosity generated during the filament fabrication. The typical void size is of the order of 50 µm, but with a large size dispersion. Thus, it can be stated that the composition of the as-received PHW filament is PLA-PHA, recycled pinewood particles, and some amount of porosity generated during the filament extrusion process.

### 3.2. Thermal Properties of as-Received and Extruded Filaments

**Figure 5:**
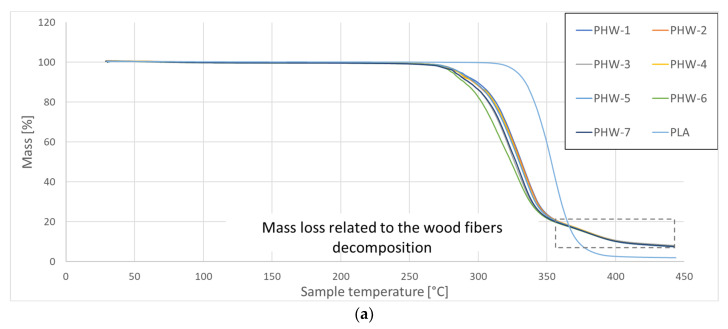

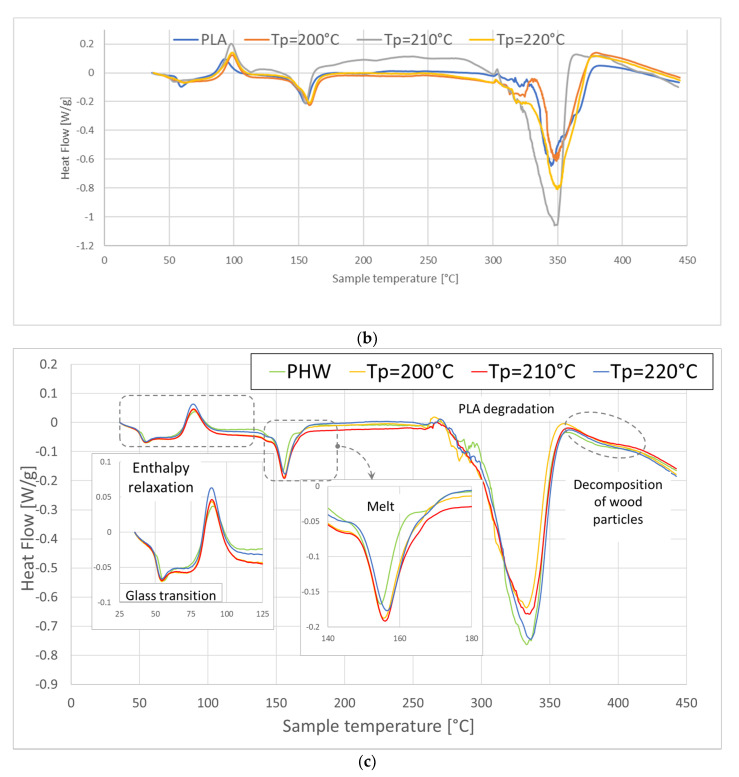
Thermal transitions assessed by DSC for as-received and extruded PHW filament at three printing temperatures (200 °C, 210 °C, and 220 °C): (**a**) mass loss kinetics for various replicates of PHW and comparison with PLA behaviour, (**b**) heat flow trends for both as-received and extruded PHW, and (**c**) heat flow trends for reference PLA.

The thermal analysis conducted using DSC shows the main thermal transitions for the as-received and extruded PHW filament at different temperatures (from 200 °C to 220 °C). Figure 5a exhibits the mass loss curves for seven replicates of the as-received PHW. In addition, the mass loss of the as-received PLA filament is plotted as a reference filament. During its heating, the main thermal changes for PLA are the glass transition and melting, which both lie within the temperature range between 50 °C and 170 °C [29]. These are further evidenced from the heat flow results for the PLA taken as a reference (Figure 5b). The main differences between the PLA and PHW filaments can be separated according to a low and high temperature range. In the low temperature range between 170 °C, modifications in the glass transition and melting are observed. In the high temperature range, framed in Figure 5a, the degradation of the wood particles clearly separates the behaviour of the two filaments at approximately 350 °C (Figure 5b,c). From this difference, it is possible to provide a precise measurement of the weight content of the wood particles in the PHW filament directly from the analysis of the weight loss. This method was originally proposed in the former work for other feedstock materials such as PLA-flax [30]. This analysis is performed using 16 replicates. It shows that the wood particle weight content is 12.96 ± 0.17% in the PHW filament. This weight content determination is very accurate, for which the scatter obtained using the 16 tested samples is only 1%. Table 4 provides the main thermal transitions, such as the glass transition, enthalpy relaxation, melting, and degradation, for the studied filament. Each column reads as one thermal transition event. Thus, for each printing condition, the five thermal events are monitored. Each event is described by four main features: enthalpy, onset, peak, and endset. From Figure 5c and the data reported in Table 4 for the as-received PHW filament, it can be stated that the thermal behaviour of the studied filament has a great stability. Indeed, the peak value for the thermal transition varies, on average, only by 1% from one replicate to another. This stability can be interpreted as being related to the homogeneity of the wood particle distribution within the filament. The glass transition identified from the position of the first peak is 56 °C for the as-received PHW filament. This temperature is lower compared to the glass transition achieved for the as-received PLA filament, which is close to 59.5 °C (Figure 5b). When the filament is extruded, a slight decrease of 4% in the glass transition temperature ($T_{g}$) is observed. However, there is no trend recorded between the $T_{g}$ and the printing temperature ($T_{P}$). $T_{g}$ stabilises at 56 °C, irrespective of the printing conditions. The next peak corresponds to the thermal relaxation at approximately 90 °C for the as-received PHW filament. This relaxation decreases by only 1% with an increase in the printing temperature. The melting temperature of the PLA follows the relaxation phenomenon, which stabilises at a relatively low temperature of 155 °C. The printing temperature has no effect on the filament melting, despite the slight increase of 1 °C in the melting temperature. The thermal degradation of the PLA within the PHW filament starts at 285 °C, with a peak value of 331 °C. The extrusion of the PHW filament shifts this temperature towards larger levels, but the amount of this change can be stated to be insignificant. The degradation of the wood particles is observed from the peak at 387 °C and continues up to 411 °C. There is, again, no significant effect of the printing temperature on the kinetics of the wood particle degradation.

### 3.3. Thermal Properties of 3D Printed Wood-PLA Filament

The thermal properties of the 3D printed wood-PLA filament are quantified using thermal conductivity (k), thermal effusivity (ω), and thermal diffusivity (τ) as a function of the printing conditions, including the printing temperature and the infill rate.

Table 5 summarises the thermal performance as a function of the infill rate (I). It is found that the thermal conductivity (k) is positively correlated with the infill rate because of the lower amount of air trapped in the printed structures with a higher infill. According to the result of the fitting procedure, the correlation is nonlinear of the form.

(1) $$k\;\left(\mathrm{mW}/\left(\mathrm{mK}\right)\right)=81-253/\left(1+exp\left(I\left(\%\right)+12.65\right)/11.6\right)\;\;\;\;\;\;\;\;\;\;\;\;\;\;\;\;\;\;\;\;\;R^{2}=1.00$$

As for thermal effusivity (ω), this is also found to be positively correlated with the infill rate, with the same nonlinear trend.

(2) $$\omega \;\left(Ws^{0.5}/\left(m^{2}K\right)\right)=234-1209/\left(1+exp\left(I\left(\%\right)+27.7\right)/14.6\right)\;\;\;\;\;\;\;\;\;\;\;\;R^{2}=1.00$$

With an opposite trend, the thermal diffusivity (τ) varies negatively with an increase in the infill rate. In this particular case, an exponential decay function truly represents the outcome of the correlation:

(3) $$\tau \;\left(\times 10^{-7}{\;m}^{2}/s\right)=1.50+1.00\times exp\left(-I\left(\%\right)/13.8\right)\;\;\;\;\;\;\;\;\;\;\;R^{2}=1.00$$

Table 6 shows the results of the thermal properties as a function of the printing temperature for a fixed infill rate of 10%.

When conducting the same fitting procedure as that for the infill rate to correlate the thermal properties with the printing temperature, the following results are achieved. As for the thermal conductivity, a linear correlation is obtained, which shows a weak dependence of the property with respect to the printing temperature.

(4) $$k\;\left(\mathrm{mW}/\left(\mathrm{mK}\right)\right)=23.65+0.13\times Tp\;\left({}^{\circ}C\right)\;\;\;\;\;\;\;\;\;\;\;\;\;\;\;\;\;\;\;\;\;R^{2}=0.89$$

The negative linear correlation suggests that the printing temperature has a slight negative effect on the thermal conductivity. This result is related to the fact that the cohesiveness of the 3D printed structure is improved by an increase in the printing temperature, which contributes to lowering the process-induced porosity. However, the rate of change in the thermal conductivity is low enough to consider that the printing temperature has a secondary contributing role to the thermal conductivity if compared to the leading position of the infill rate.

As for the thermal effusivity (ω), the effect of the printing temperature is negligible, with an average value of 128 Ws^0.5^/(m^2^K). If a linear fitting procedure is forced, a slightly positive trend is found, but with a low correlation factor.

(5) $$\omega \;\left(Ws^{0.5}/\left(m^{2}K\right)\right)=122+0.027\times T_{P}\;\left({}^{\circ}C\right)\;\;\;\;\;\;\;\;\;\;\;\;\;\;\;\;\;\;\;\;R^{2}=0.49$$

The same opposing trend of thermal diffusivity (τ) is found with respect to the thermal conductivity. It is also found to increase with an increase in the printing temperature. The same minor effect of the printing temperature is observed compared to the infill rate. In this case, a linear trend represents the most correlation between the thermal diffusivity and the printing temperature:

(6) $$\tau \;\left(\times 10^{-7}{\;m}^{2}/s\right)=2.65-0.003\times T_{P}\;\left({}^{\circ}C\right)\;\;\;\;\;\;\;\;\;\;R^{2}=0.99$$

In summary, as shown in Table 5 and the derived equations, the thermal conductivity and effusivity are found to be positively correlated with the infill rate and printing temperature, while the opposite is found for the thermal diffusivity. This large dependence on the infill rate can be understood thanks to the large contrast between the thermal conductivity of air and that of the solid phase mainly composed of PLA. When reducing the density of the material by creating a controlled porous structure, the overall thermal conductivity of the entire sample drops, with a rate dependent on the ratio between the thermal conductivities of the phases. In our case, this rate represents 7.6 in favour of the airy phase.

Table 7 shows the results of the thermal properties of the variety of insulators used in the building sector. In terms of the thermal performance, the thermal conductivity of the 3D printed PHW is slightly larger than that of the benchmarking insulators. The performance mismatch represents about 38% with respect to synthetic foam and even 57% for glass wool. A similar mismatch in the thermal diffusivity is observed, with 53% and 51% for both synthetic foam and glass wool, respectively. For the thermal effusivity, this mismatch reaches 80% and 127% for the same benchmarked materials. The wood-PLA printed material thus has a lower insulation performance compared to glass wool and synthetic foam. However, Wood-PLA printed with an infill rate of 10% is considered as an insulator. Indeed, its thermal conductivity is below 60 mW/(m·K), which is the standard admitted to the quality of a material as an insulator in the building sector, according to the norm UNE-EN 12667:2002. The thermal performance of PHW can be, in addition, further improved by shifting the operating condition window towards lower infill rates to decrease the thermal conductivity, while preserving a good stiffness. Insulators in the building industry are known to have a low stiffness, which can be an issue, especially during the lifetime where an expected loss of thermal insulation occurs due to various reasons, such as built-in conditions and aging, etc. [31,32].

### 3.4. Mechanical Performance of as-Received and Printed Wood-PLA Material

**Figure 6:**
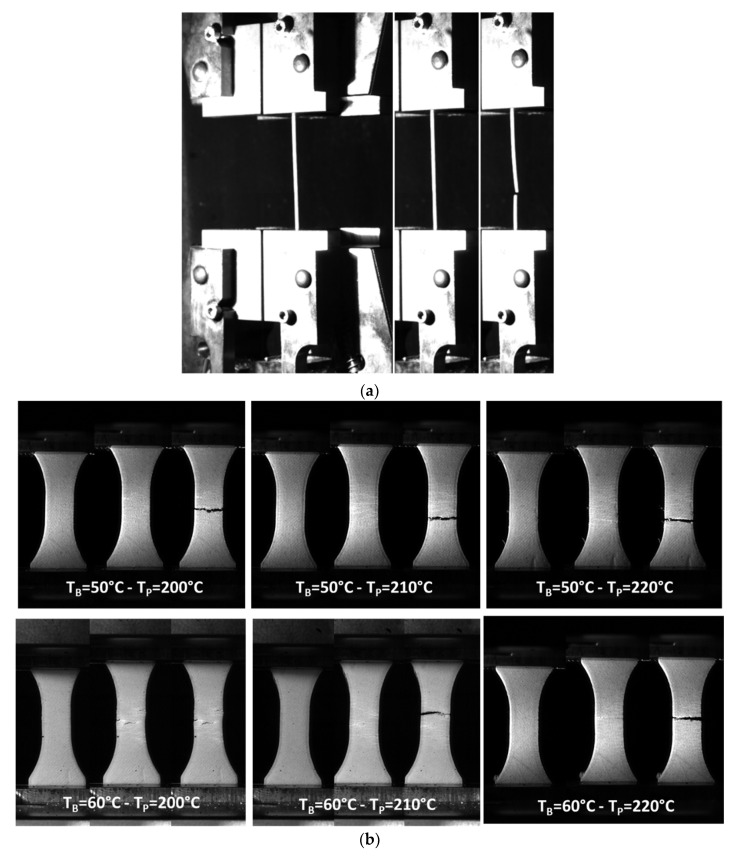

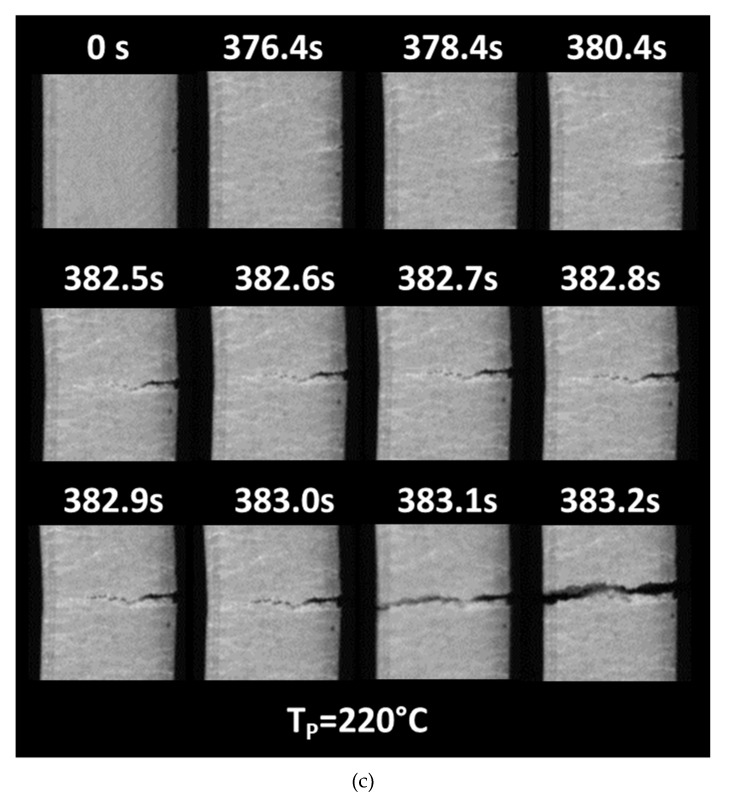
Monitoring of deformation sequence using optical imaging: (**a**) as-received PHW filament, (**b**,**c**) 3D printed PHW as a function of the printing and base temperature, and (**c**) sequence showing crack initiation and propagation up to the rupture point.

Figure 6a shows a typical deformation sequence of the as-received PHW filament. The sequence highlights the limited stretching capabilities of the filament. This behaviour contrasts with that of the PLA filament, where the amount of deformation exceeds 10%. Figure 6b compares the loading sequences of the 3D printed PHW material as a function of the printing temperature (T_P_) and a fixed base temperature (T_B_). All the samples exhibit similar limited stretching as that in the case of the as-received filament. In addition, near the rupture point, clear areas in the form of stretching bands occur, indicating damage accumulation. These areas extend along the gauge length. At the rupture point, a predominant opening mode is the main mechanism for material failure, which seems to be independent of the T_P_. The same overall behaviour is noticed for an increase in the base temperature (T_B_) to 60 °C, as shown in Figure 6c. The only difference seems to be related to the lowest T_P_, where the damage growth seems to be slower compared to that in other conditions. A further analysis of the cracking behaviour is evidenced in Figure 6c, where the optical recording is conducted at higher frame rates. A close look at the gauge length at nearly a 2-millisecond interval shows that the spots of crack departures are likely to be on the surfaces of the specimens. Indeed, despite the presence of an external frame, most of the cracks originate from the sample ends. The crack departure is subsequent to a strain localisation materialised by white bands, which tend to be more or less aligned with the filament layups. The crack growth exhibits a limited jaggedness, which may be explained by the tendency to align with the filament direction. In terms of the speed of the crack growth, there is no direct relationship between the printing conditions and the observed kinetics. Within the same sample, this crack growth may experience different phases of deceleration and acceleration, as shown in Figure 6c. The crack speed ranges, in average, between 0.97 mm/s and 34.9 mm/s.

**Figure 7:**
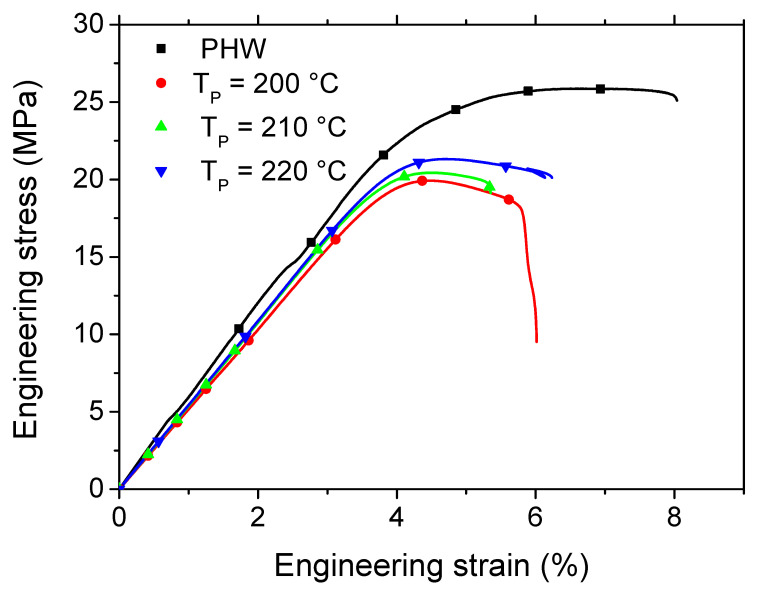
Tensile performance of as-received and 3D printed PLA-wood material as a function of the printing temperature T_P_ for a fixed base temperature T_B_.

Figure 7 shows the typical loading response of the as-received and printed PHW materials. All the materials exhibit an elastic-plastic behaviour with limited stretching. A reduction in the elongation at break is also noticed as a consequence of the printing process. In addition, as is a tendency towards a reduction in the tensile strength. However, the stiffness reduction seems to be less important compared to the effect of the printing temperature on the tensile strength. Table 8 summarises the results obtained for all the printing conditions, including the printing (T_P_) and base (T_B_) temperatures. In the same Table, the mechanical properties of the as-received PLA are also reported, based on data from a previous study [33]. A large drop is observed in the performance when blending the PLA with wood particles. With the exception of elongation at break, most of the engineering constants are halved in magnitude. This drop in performance is explained by the presence of porosity within the wood-PLA filament. Furthermore, a weak interface between the PLA matrix and wood particles can also lead to a decline in the filament performance. An analysis of the Young’s modulus results of the 3D printed PHW confirm the limited sensitivity of this engineering constant to the printing conditions, including the printing and base temperatures. These results are achieved with a good confidence, where the ratio between the standard deviation and average value is only 2.3%. Also, the limited loss of stiffness due to the printing process is confirmed. This loss represents, on average, 1%, and indicates that the generated porosity has a negligible effect on the stiffness of the 3D printed PHW. In other words, both the printing and base temperatures have no significant effect on the stiffness within the considered parameter window. The yield stress values have no marked tendency with respect to the printing and base temperatures. The ratio between the standard deviation and average values of the yield stress (3.8%) is lower compared to the Young’s moduli data. The amount of loss compared to the filament properties is 14%. Slightly large tensile strength values seem to be related to an increase in the printing temperature. The amount of performance loss is similar to the case of yield stress, which is of the order of 19% compared to the filament performance, irrespective of the printing and base temperatures. Similar observations about the insensitivity to the printing conditions are confirmed in the cases of the elongation at break and ultimate stress. These two engineering constants trigger the most significant loss in performance, with 29% and 25% decreases compared to the filament properties. In summary, the mechanical properties of the printed wood-PLA, including its Young’s modulus, yield stress, tensile strength, ultimate stress, and elongation at break, are found to be insensitive to the printing temperature.

**Figure 8:**
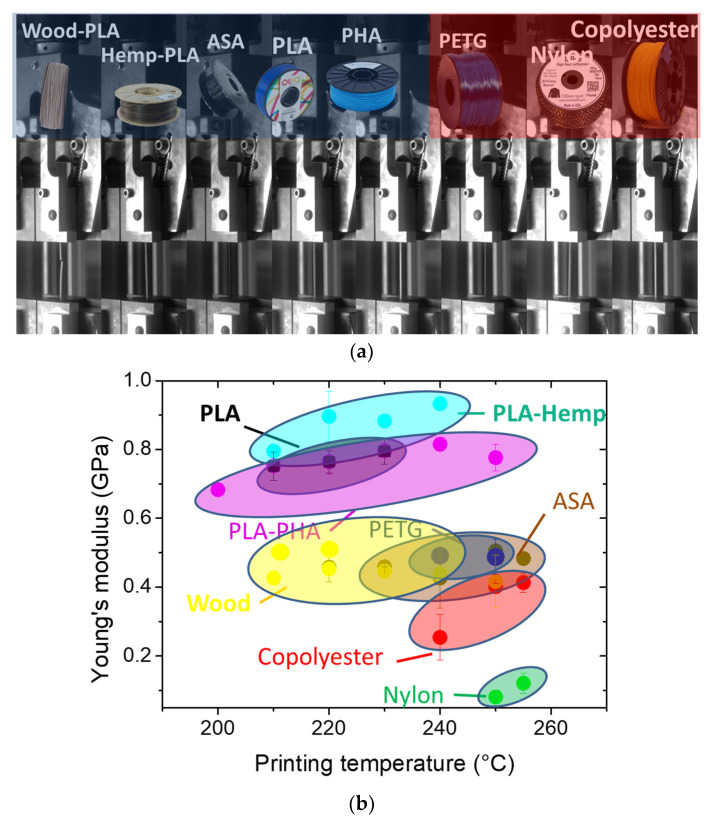

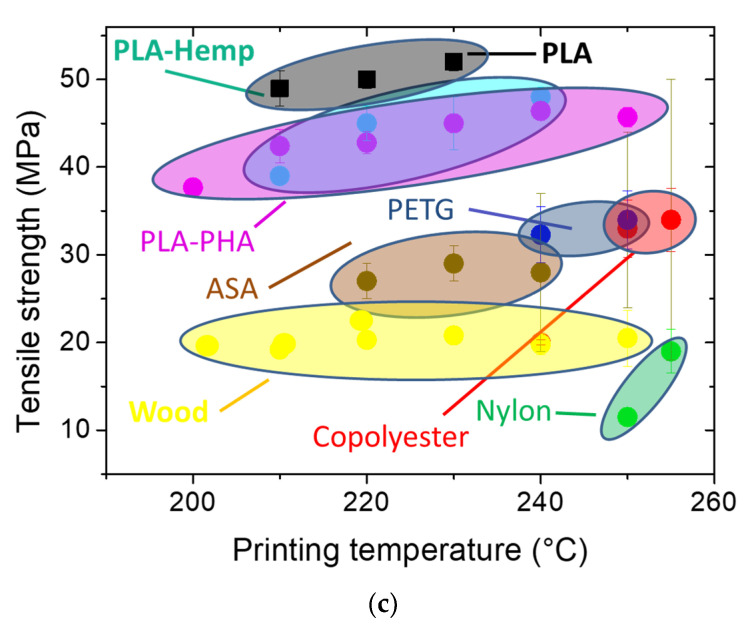
Comparison between the mechanical performance of wood-PLA and a collection of feedstock materials for fused filament as a function of the printing temperature: (**a**) deformation sequences showing the ranking of the elongation at break of as-received filaments, (**b**) Young’s modulus, and (**c**) tensile strength of 3D printed materials as a function of the printing temperature.

Figure 8 compares the performances of several feedstock materials, including wood-PLA materials. Figure 8a illustrates the tensile response of the as-received filaments for a collection of eight materials. Among the filaments compared, copolyester, nylon, and PETG reach the largest elongation at break. Composite materials such as wood-PLA and hemp-PLA are positioned towards the lower end of the ranking. Figure 8b depicts the predicted stiffness performances of the printed dogbones for the same collection of feedstock materials. These are tested under the same conditions as the wood-PLA printed material and compared according to previous research works by the authors [33]. The stiffness of the wood-PLA printed material is in the mid-range compared to the remaining feedstock materials printed within the same printing temperature window. Figure 8c shows that the ranking of the wood-PLA material is lower compared to its stiffness. With respect to the other feedstock materials, wood-PLA stands at the second position after nylon for the tensile strength. It has to be mentioned that the printing temperatures considered for nylon are well bellow the standards. Indeed, the large amount of porosity generated by the process conditions is larger than the other materials.

### 3.5. Filament Arrangement and Fracture Patterns

**Figure 9:**
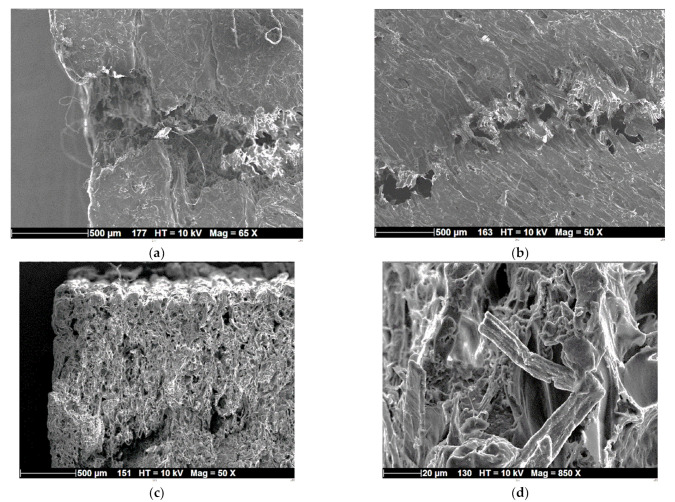
SEM micrographs showing the fracture patterns at different magnifications and positions: (**a**) crack initiation site, (**b**) crack within the raster, (**c**) cross-section view of the crack pattern, and (**d**) zoomed view close to a wood particle.

Figure 9 shows typical SEM micrographs of the fractured samples, which detail the main characteristics of the fracturing behaviour of the 3D printed PHW samples. Figure 9a depicts one of the initiation sites for the crack propagation. The micrograph shows that the crack departure is subsequent to a rupture in the external frame. This frame provides mechanical stability to the inner core of the PHW prints. Figure 9b shows a zoomed view along the crack path within the raster, where the underlined microstructure near the jagged crack is revealed. The decohesion between adjacent filaments seems to be the origin of this jaggedness, where the crack tends to follow a path of minimum energy connecting the space between adjacent filaments. However, a large deviation in the crack is prevented by the multiplicity of the defects ahead of the crack, which forces the crack to follow a predominant opening mode. These defects are illustrated in Figure 9c through the cross-section view, where globular porosities are depicted with typical sizes from 50 to 70 µm. These porosities are identified as genuine to the as-received filament in Figure 4c. Figure 9d depicts a zoomed view near a ruptured wood pine fibre with a typical diameter of the order of 12 µm. This micrograph reveals some of the rupture mechanisms occurring at the scale of the filament reinforcement. Tensile failure due to excessive elongation, as well as bending at weak points, are the main observed mechanisms at a relatively small scale within the filament. These mechanisms compete with other mechanisms happening at much larger scales (100 µm, 1 mm), where the stress concentrators near the globular porosity activate more complex tension/shear deformation and further extend in the raster and at the external frame.

Thus, it can be stated that the pores generated by the process act as stress concentrators. The particular feature of AM is that it generates highly connected porosities, which tend to connect through a crack percolation mechanism, as shown in a former study by the group using an X-ray micro-tomography analysis [34].

The overall tensile loading is transferred to the structure according to two main mechanisms:

- Pure uniaxial tension, which localizes at the periphery. This mechanism is related to the presence of an external frame that promotes uniaxial deformation in the loading direction
- Combined uniaxial and shear deformation, which is related to the misalignment of the filament within the raster, with respect to the loading conditions.

The two former mechanisms are in-plane. When the building direction is aligned with the loading direction, another mechanism leads to the failure of the material, according to the mechanism of layer delamination.

No marked differences are observed between the porous structures observed for the fully dense materials printed at different printing temperatures. This can explain the insensitivity of the mechanical properties to the printing temperature.

## 4. Conclusions

The conclusion of this study concerns two main aspects: from a mechanical viewpoint, this study showed that wood-PLA filaments encompass a large structural complexity related to the presence of inner porosity within the filaments. When printed, this material exhibited a limited loss in its mechanical performance compared to the filaments’ properties, which materialised by decreases of 1%, 19%, 25%, and 29% for the Young’s modulus, tensile strength, ultimate stress, and elongation at break. This means that wood-PLA can be used as a rigid insulator, because this loss in performance was not significant, especially when looking at the stiffness results. Besides the loss in performance, the wood-PLA deformation mechanisms did not relate much to the filament arrangements. For instance, the study concludes that the predominant opening mode still prevails, which is explained by the multiplicity of the defects, especially the porosity within of the as-received filament. 

The second main conclusion is that wood-PLA’s thermal properties can be controlled with AM, resulting in an insulation material that can be used in the building sector. In fact, the thermal performance of the printed wood-PLA material showed a significant trend with the infill rate, and only a limited effect was found for the printing temperature. Indeed, the study showed that the thermal conductivity decreased by 37% when the infill rate went from 40% down to 10%. The thermal diffusivity and effusivity followed similar trends, with 21% and 43% improvement, respectively. Within the operating condition window, the achieved thermal performance, especially at 10% of infill rate, was still not satisfactory. There was, however, room for improvement by further decreasing the infill rate. The thermal conductivity of the 3D printed wood-PLA for an infill rate of 10% qualifies the material to be an insulator in the building sector, according to the standard UNE-EN 12667:2002.

In terms of the potential of using this material as an insulator in the building sector, its low thermal performance compared to materials such as foams and wools does not come as a favourable argument, although thermal conductivities as low as 50 mW/(mK) were observed for an infill rate of 10%. The gaps of the thermal performance represent 57% and 38% with respect to glass wool and synthetic foams. To make it more competitive with synthetic insulators, the infill rate of wood-PLA should be further decreased. However, the downside of this is the risk of lowering the mechanical stability of wood-PLA printed material.

Finally, it can be stated that the combination of its thermal insulation and mechanical performance makes wood-PLA material a good candidate to ensure both structural stability and insulating functions.

## Figures and Tables

**Table 1:** Physical properties and printing conditions of and PHW feedstock material.

Property	Level
Wire diameter, (mm)	1.75 ± 0.05 mm
Density, (g/cm^3^)	1.15 g/cm^3^ (ISO 1183) [23]
Tensile modulus, (MPa)	3290 (ISO 527) [24]
Tensile strength, (MPa)	46 (ISO 527)
Tensile strain at tensile strength, (%)	4.6 (ISO 527)
Tensile stress at break, (MPa)	42 (ISO 527)
Tensile strain at break, (MPa)	5.5 (ISO 527)
Flexural modulus, (MPa)	3930 MPa (ISO 178) [25]
Flexural strain at break, (%)	5% (ISO 178)
Sharpy notched impact strength, (kJ/m^2^)	4.2 (ISO 179-1/1 eA) [26]
Sharpy impact strength, (kJ/m^2^)	19.0 (ISO 179-1/1 eU)
Glass transition temperature, °C	55 °C
Printing temperature, °C	195–220 °C
Bed temperature, °C	50–60 °C
Printing speed, mm/s	40–100

**Table 2:** Fixed printing conditions of PHW feedstock material.

Printing Condition	Value
Nozzle diameter, mm	0.4
Layer height, mm	0.2
Wall thickness, mm	0.8
Top/bottom thickness, mm	0.6
Printing speed, mm/s	50
Building sequence, deg.	+45/−45

**Table 3:** Parameters used to assess thermal properties of 3D printed PHW and commercial insulators: source power (W)/test duration (s)/heating duration (s).

Material	Thermal Conductivity	Thermal Effusivity	Thermal Diffusivity
3D printed PHW	0.14/50/0	2.1/50/0	1.5/100/30
Glass wool	0.1/30/0	1/30/0	1/65/25
Synthetic foam	0.8/35/0	1/30/0	0.61/60/25

**Table 4:** Thermal transitions in PLA and PHW as function of printing conditions. Thermal transitions are identified by E: Enthalpy (J·g^−1^); O: Onset (°C); P: Peak (°C); and E: Endset (°C).

Material	Thermal Transitions: E/O/P/E				
PHW—as-received	3.8 ± 0.92 51 ± 0.8 56 ± 0.4 63 ± 1.1	13.9 ± 0.40 80 ± 0.5 90 ± 0.5 103 ± 0.7	20.8 ± 0.66 147 ± 0.5 155 ± 0.2 161 ± 0.3	310.2 ± 17.24 285 ± 10.6 331 ± 2.9 353 ± 4.9	5.5 ± 1.05 264 ± 1.2 387 ± 1.5 411 ± 1.6
PHW—T_P_ = 200 °C	3.1 ± 0.25 49 ± 0.4 54 ± 0.3 62 ± 1.0	15.3 ± 0.85 80 ± 0.2 90 ± 0.2 102 ± 0.4	21.7 ± 1.01 148 ± 0.3 156 ± 0.5 164 ± 0.6	318.8 ± 5.71 277 ± 3.3 334 ± 1.0 355 ± 1.6	4.1 ± 0.39 364 ± 2.9 386 ± 0.9 411 ± 1.0
PHW—T_P_ = 210 °C	3.4 ± 0.12 49 ± 0.0 54 ± 0.3 62 ± 0.4	16.5 ± 0.13 80 ± 0.3 89 ± 0.4 102 ± 0.9	21.4 ± 0.55 149 ± 0.1 156 ± 0.2 164 ± 0.7	336.1 ± 46.83 279 ± 1.0 335 ± 1.5 357 ± 0.3	3.8 ± 0.65 368 ± 1.1 385 ± 1.4 411 ± 1.3
PHW—T_P_ = 220 °C	3.2 ± 0.27 49 ± 0.1 54 ± 0.1 62 ± 0.3	16.2 ± 0.18 81 ± 0.2 89 ± 0.1 101 ± 0.5	23.1 ± 1.06 149 ± 0.2 156 ± 0.4 164 ± 0.4	328.6 ± 5.34 284 ± 0.4 335 ± 1.8 358 ± 1.8	2.6 ± 1.50 368 ± 0.7 385 ± 0.6 407 ± 5.9

**Table 5:** Thermal performance of 3D printed PHW as a function of the infill rate for a fixed printing temperature (T_P_ = 200 °C). STD is the standard deviation.

Property	Infill Rate (%)			
Infill rate (%)	10	20	30	40
Density (kg/m^3^)	168 ± 6	261 ± 1	333 ± 10	399 ± 2
Thermal conductivity (mW/(mK))	49.50 ± 1.29	66.75 ± 1.43	73.25 ± 2.99	78.75 ± 2.36
Thermal effusivity (Ws^0.5^/(m^2^K))	127 ± 4	179 ± 1	199 ± 4	221 ± 3
Thermal diffusivity (×10^−7^ m^2^/s)	1.98 ± 0.02	1.74 ± 0.05	1.59 ± 0.06	1.56 ± 0.05

**Table 6:** Thermal performance of 3D printed PHW as a function of the printing temperature (T_P_) for a fixed infill rate (10%).

Property	Printing Temperature (°C)		
	200	210	220
Thermal conductivity (mW/(mK))	49.50 ± 1.29	50.25 ± 0.96	52.25 ± 1.71
Thermal effusivity (Ws^0.5^/(m^2^K))	127 ± 4	128 ± 2	128 ± 1
Thermal diffusivity (×10^−7^ m^2^/s)	1.98 ± 0.02	1.95 ± 0.01	1.91 ± 0.03

**Table 7:** Thermal performance of tested insulators.

Property	Material	
Material	Glass wool	Synthetic Foam
Density (kg/m^3^)	106 ± 0.00	31.3
Thermal conductivity (mW/(mK))	31.50 ± 1.00	36.00 ± 1.41
Thermal effusivity (Ws^0.5^/(m^2^K))	61.00 + 1.15	70.50 ± 1.29
Thermal diffusivity (×10^−7^ m^2^/s)	4.08 ± 0.06	4.23 ± 0.05

**Table 8:** Mechanical performance of 3D printed PHW as a function of printing temperature.

Material	T_B_ (°C)	T_P_ (°C)	E_Y_ (GPa)	σ_Y_ (MPa)	σ_S_ (MPa)	σ_R_ (MPa)	ε_R_ (%)
PLA	-	-	1.09 ± 0.14	42.56 ± 3.82	54 ± 0.00	47.83 ± 0.77	5.5 ± 1.8
PHW	-	-	0.54 ± 0.058	24.55 ± 0.481	25.53 ± 0.319	21.84 ± 2.539	7.6 ± 0.8
3D printed	50	200	0.53 ± 0.010	18.71 ± 0.988	20.60 ± 1.101	18.65 ± 1.987	5.3 ± 1.99
	60	200	0.53 ± 0.015	18.88 ± 0.524	20.70 ± 0.680	16.23 ± 5.834	5.6 ± 0.40
	50	210	0.53 ± 0.005	18.56 ± 0.589	20.39 ± 0.672	17.56 ± 0.075	5.6 ± 0.30
	60	210	0.53 ± 0.013	18.33 ± 0.883	20.12 ± 1.028	17.02 ± 3.681	5.6 ± 0.30
	50	220	0.53 ± 0.021	18.72 ± 1.078	20.62 ± 1.097	15.92 ± 5.515	6.1 ± 0.3
	60	220	0.54 ± 0.012	18.97 ± 0.229	21.12 ± 0.285	18.66 ± 2.058	6.2 ± 0.1

## Data Availability

Data are available from the authors on request.
